# Supplementary material for: Molecular orbital theory in cavity QED environments
Source: Nat Commun. 2022 Mar 15;13:1368. doi: 10.1038/s41467-022-29003-2 (PMC8924263; doi:10.1038/s41467-022-29003-2)
Supplement: Supplementary file 1 — Supplementary Information [file 41467_2022_29003_MOESM1_ESM.pdf]

Supplementary Information:
Molecular orbital theory in cavity QED environments

H. Koch et al.

Supplementary Methods

SC-QED-HF theory

The light-matter interaction is modeled using the Pauli-Fierz dipole Hamiltonian in length gauge

$$H = H_e + \omega b^\dagger b + \lambda\sqrt{\frac{\omega}{2}}(\mathbf{d}\cdot\boldsymbol{\epsilon})(b+b^\dagger) + \frac{\lambda^2}{2}(\mathbf{d}\cdot\boldsymbol{\epsilon}), \tag{1}$$

where $\mathbf{d}$ is the molecular dipole operator, $\boldsymbol{\epsilon}$ is the field polarization, $\lambda = \frac{1}{\sqrt{\epsilon_0 V}}$ is the coupling constant between the molecule and the field and V is the quantization volume. The frequency of the electric field is given by ω, and the bosonic operators $b^\dagger$ and b respectively create and annihilate photons. The electronic Hamiltonian H_e is given by the expression

$$H_e = \sum_{pq} h_{pq} E_{pq} + \frac{1}{2}\sum_{pqrs} g_{pqrs} e_{pqrs}, \tag{2}$$

where the following definitions have been used:

$$\begin{aligned}
h_{pq} &= \int \phi_p^*(r)\left(-\frac{\nabla^2}{2} - \sum_I \frac{Z_I}{|r-R_I|}\right)\phi_q(r) d^3r \\
g_{pqrs} &= \int\int \phi_p^*(r)\phi_r^*(r')\frac{1}{|r-r'|}\phi_q(r)\phi_s(r') d^3r \\
E_{pq} &= \sum_\sigma a_{p\sigma}^\dagger a_{q\sigma} \\
e_{pqrs} &= \sum_{\sigma\tau} a_{p\sigma}^\dagger a_{r\tau}^\dagger a_{s\tau} a_{q\sigma}
\end{aligned} \tag{3}$$

In Equations (3), I labels the nuclei and $a_{p\sigma}^\dagger$ and $a_{p\sigma}$ respectively create and destroy an electron in orbital p with spin σ. In the infinite coupling limit $\lambda \longrightarrow \infty$, the electron-photon interaction dominates the electronic contributions. We therefore only consider the photonic part of the Hamiltonian when determining the eigenfunctions

$$H_\infty = \omega b^\dagger b - \lambda\sqrt{\frac{\omega}{2}}(\mathbf{d}\cdot\boldsymbol{\epsilon})(b+b^\dagger) + \frac{\lambda^2}{2}(\mathbf{d}\cdot\boldsymbol{\epsilon})^2. \tag{4}$$

Since the dipole operator is the only electronic operator in this Hamiltonian, the electronic part of an eigenfunction will be a Slater determinant in the orbitals basis that diagonalizes the dipole. The form of the eigenfunctions is given as:

$$|\psi\rangle = \exp\left(-\frac{\lambda}{\sqrt{2\omega}}\sum_p (\mathbf{d}\cdot\boldsymbol{\epsilon})_{pp} E_{pp}(b-b^\dagger)\right)\prod_i^{n_{occ}} a_i^\dagger |\text{vac}\rangle \otimes |0\rangle, \tag{5}$$

where $|\text{vac}\rangle$ is the vacuum for the electrons and $|0\rangle$ is the photonic vacuum. We highlight that in the infinite coupling limit every orbital p has its own coherent state parameter which is determined by the eigenvalues of $\mathbf{d}\cdot\boldsymbol{\epsilon}$,

$(\mathbf{d}\cdot\boldsymbol{\epsilon})_{pp}$. Transforming the full Hamiltonian to the dipole basis, we obtain

$$\begin{aligned}\tilde{H} =&\tilde{H}_e + \omega b^\dagger b - \lambda\sqrt{\frac{\omega}{2}}\sum_p (\mathbf{d}\cdot\boldsymbol{\epsilon})_{pp} E_{pp}(b+b^\dagger) + \frac{\lambda^2}{2}\sum_{pq} (\mathbf{d}\cdot\boldsymbol{\epsilon})_{pp} (\mathbf{d}\cdot\boldsymbol{\epsilon})_{qq} e_{ppqq} \\ &+ \frac{\lambda^2}{2}\sum_p (\mathbf{d}\cdot\boldsymbol{\epsilon})_{pp} (\mathbf{d}\cdot\boldsymbol{\epsilon})_{pp} E_{pp},\end{aligned} \tag{6}$$

where $\tilde{H}_e$ is equal to

$$\tilde{H}_e = \sum_{pq} \tilde{h}_{pq} E_{pq} + \frac{1}{2}\sum_{pqrs} \tilde{g}_{pqrs} e_{pqrs} \tag{7}$$

with $\tilde{h}_{pq}$ and $\tilde{g}_{pqrs}$ are defined as:

$$\begin{aligned}\tilde{h}_{pq} &= \sum_{rs} U_{rp} h_{rs} U_{sq} \\ \tilde{g}_{pqrs} &= \sum_{mntu} U_{mp} U_{tr} g_{mntu} U_{nq} U_{us}\end{aligned} \tag{8}$$

and U is the matrix the rotates from to the basis that diagonalizes the dipole operator. The SC-QED-HF wave function is obtained by relaxing the infinite coupling solutions in Equation (5). In particular we allow for:

- A variationally optimized orbital specific coherent coefficient η_p.
- A unitary rotation in between the mixed electron photon operator and the $|\mathrm{HF}\rangle$ state, meaning that the dipole basis will not be the canonical basis.

We define the SC-QED-HF wave function

$$|\psi\rangle = \exp\left(-\frac{\lambda}{\sqrt{2\omega}}\sum_p \eta_p E_{pp}(b-b^\dagger)\right) \exp(\kappa) \prod_i^{n_{occ}} a_i^\dagger |\mathrm{vac}\rangle \otimes |0\rangle\,, \tag{9}$$

where the E_{pp} is in the dipole basis and κ is an antihermitian operator used to parametrize a unitary rotation that will be variationally optimized

$$\kappa = \sum_{p>q} k_{pq}(E_{pq} - E_{qp}). \tag{10}$$

The energy obtained using the parametrization in Equation (9) is:

$$\begin{aligned}E =& \sum_{pq} \tilde{h}_{pq} D_{pq} \exp\left(-\frac{\lambda^2 (\eta_p - \eta_q)^2}{4\omega}\right) \\ &+ \frac{\lambda^2}{2}\sum_p D_{pp} \left((\mathbf{d}\cdot\boldsymbol{\epsilon})_{pp} - \eta_p\right)\left((\mathbf{d}\cdot\boldsymbol{\epsilon})_{pp} - \eta_p\right) \\ &+ \frac{\lambda^2}{2}\sum_{pq} \left(D_{pp}D_{qq} - \frac{D_{pq}D_{qp}}{2}\right)\left((\mathbf{d}\cdot\boldsymbol{\epsilon})_{pp} - \eta_p\right)\left((\mathbf{d}\cdot\boldsymbol{\epsilon})_{qq} - \eta_q\right) \\ &+ \frac{1}{2}\sum_{pqrs} \tilde{g}_{pqrs}\left(D_{pq}D_{rs} - \frac{D_{ps}D_{rq}}{2}\right) \exp\left(-\frac{\lambda^2 (\eta_p + \eta_r - \eta_q - \eta_s)^2}{4\omega}\right),\end{aligned} \tag{11}$$

where $D_{pq} = \langle \mathrm{HF}|\ e^{-\kappa} E_{pq}\ e^{\kappa}|\mathrm{HF}\rangle$ are the density matrix elements in the dipole basis and we note that the contributions from $\tilde{H}_e$ are scaled with Gaussians factors. To derive Equation (11) we used the following relation:

$$\exp\left(\frac{\lambda}{\sqrt{2\omega}}\sum_n \eta_n E_{nn}(b-b^\dagger)\right) a_p^\dagger \exp\left(-\frac{\lambda}{\sqrt{2\omega}}\sum_n \eta_n E_{nn}(b-b^\dagger)\right) = a_p^\dagger \exp\left(\frac{\lambda\eta_p}{\sqrt{2\omega}}\left(b-b^\dagger\right)\right), \tag{12}$$

The variational optimization of the η_p and κ parameters is achieved using an energy minimization procedure. In particular, the optimal parameters are obtained when the energy gradients with respect to the η_p and D_{pq} parameters are equal to zero.

$$
\begin{aligned}
\frac{\partial E}{\partial D_{mn}} &= \tilde{h}_{mn} Q_{mn} + \sum_{rs} \left(2\tilde{g}_{mnrs} - \tilde{g}_{msrn}\right) D_{rs} Q_{mnrs} \\
&+ \frac{\lambda^2}{2} \delta_{mn} \left((\mathbf{d}\cdot\boldsymbol{\epsilon})_{mm} - \eta_m\right)^2 \\
&- \lambda^2 D_{nm} \left((\mathbf{d}\cdot\boldsymbol{\epsilon})_{mm} - \eta_m\right)\left((\mathbf{d}\cdot\boldsymbol{\epsilon})_{nn} - \eta_n\right) \\
&+ 2\lambda^2 \delta_{mn} \left((\mathbf{d}\cdot\boldsymbol{\epsilon})_{mm} - \eta_m\right) \sum_q D_{qq} \left((\mathbf{d}\cdot\boldsymbol{\epsilon})_{qq} - \eta_q\right) \\
\frac{\partial E}{\partial \eta_m} &= \frac{\lambda^2}{\omega} \sum_q \tilde{h}_{mq} D_{mq} \left(\eta_q - \eta_m\right) Q_{mq} - \lambda^2 D_{mm} \left((\mathbf{d}\cdot\boldsymbol{\epsilon})_{mm} - \eta_m\right) \\
&+ \frac{\lambda^2}{\omega} \sum_{qrs} \tilde{g}_{mqrs} \left(D_{mq} D_{rs} - \frac{D_{ms} D_{rq}}{2}\right) \left(\eta_m + \eta_r - \eta_q - \eta_s\right) Q_{mqrs} \\
&- \lambda^2 \sum_q \left(D_{qq} D_{mm} - \frac{D_{qm}^2}{2}\right) \left((\mathbf{d}\cdot\boldsymbol{\epsilon})_{qq} - \eta_q\right)
\end{aligned}
\tag{13}
$$

where Q_{pq} and Q_{pqrs} are defined as:

$$
Q_{pq} = \exp\left(-\frac{\lambda^2 \left(\eta_p - \eta_q\right)^2}{4\omega}\right) \tag{14}
$$

$$
Q_{pqrs} = \exp\left(-\frac{\lambda^2 \left(\eta_p + \eta_r - \eta_s - \eta_q\right)^2}{4\omega}\right). \tag{15}
$$

We notice that at a stationary point where the gradients are equal to zero the following relation holds:

$$
\begin{aligned}
\sum_m \frac{\partial E}{\partial \eta_m} &= -\lambda^2 \sum_{mq} \left(D_{qq} D_{mm} - \frac{D_{mq}^2}{2}\right) \left((\mathbf{d}\cdot\boldsymbol{\epsilon})_{qq} - \eta_q\right) - \lambda^2 \sum_m D_{mm} ((\mathbf{d}\cdot\boldsymbol{\epsilon})_{mm} - \eta_m) \\
&= \sum_m D_{mm} ((\mathbf{d}\cdot\boldsymbol{\epsilon})_{mm} - \eta_m) = 0,
\end{aligned}
\tag{16}
$$

which implies

$$
\sum_m D_{mm}\, \eta_m = \langle \mathbf{d}\cdot\boldsymbol{\epsilon} \rangle. \tag{17}
$$

Total energy and orbital energy origin invariance

In this section we demonstrate the origin invariance of both the total energy and the individual orbital energies for SC-QED-HF. Assume that we have found an optimal set of parameters $\eta_p = \bar{\eta}_p$, $D_{pq} = \bar{D}_{pq}$, such that

$$
\frac{\partial E(D_{pq}, \omega_p)}{\partial D_{pq}} |_{\bar{D}_{pq}, \bar{\eta}_p} = 0 \tag{18}
$$

$$
\frac{\partial E(D_{pq}, \eta_p)}{\partial \eta_p} |_{\bar{D}_{pq}, \bar{\eta}_p} = 0. \tag{19}
$$

If the system is displaced in space by a vector $\mathbf{a}$, the dipole matrix elements will change according to:

$$
(\mathbf{d}\cdot\boldsymbol{\epsilon})_{pq} \longrightarrow (\mathbf{d}\cdot\boldsymbol{\epsilon})_{pq} + \frac{Q_{tot}}{N_e} (\mathbf{a}\cdot\boldsymbol{\epsilon}) \delta_{pq}, \tag{20}
$$

where N_e is the number of electrons and Q_{tot} is the total charge of the system. Therefore the energy expression in Equation (11) takes the form

$$
\begin{aligned}
E =& \sum_{pq} \tilde{h}_{pq} \bar{D}_{pq} \exp\left(-\frac{\lambda^2 \left(\bar{\eta}_p - \bar{\eta}_q\right)^2}{4\omega}\right) \\
&+ \frac{\lambda^2}{2} \sum_{p} \bar{D}_{pp} \left((\mathbf{d}\cdot\boldsymbol{\epsilon})_{pp} + \frac{Q_{tot}}{N_e}(\mathbf{a}\cdot\boldsymbol{\epsilon}) - \bar{\eta}_p\right)\left((\mathbf{d}\cdot\boldsymbol{\epsilon})_{pp} + \frac{Q_{tot}}{N_e}(\mathbf{a}\cdot\boldsymbol{\epsilon}) - \bar{\eta}_p\right) \\
&+ \frac{1}{2} \sum_{pqrs} \tilde{g}_{pqrs} \left(\bar{D}_{pq}\bar{D}_{rs} - \frac{\bar{D}_{ps}\bar{D}_{rq}}{2}\right) \exp\left(-\frac{\lambda^2 \left(\bar{\eta}_p + \bar{\eta}_r - \bar{\eta}_q - \bar{\eta}_s\right)^2}{4\omega}\right) \\
&+ \frac{\lambda^2}{2} \sum_{pq} \left(\bar{D}_{pp}\bar{D}_{qq} - \frac{\bar{D}_{pq}\bar{D}_{qp}}{2}\right)\left((\mathbf{d}\cdot\boldsymbol{\epsilon})_{pp} + \frac{Q_{tot}}{N_e}(\mathbf{a}\cdot\boldsymbol{\epsilon}) - \bar{\eta}_p\right)\left((\mathbf{d}\cdot\boldsymbol{\epsilon})_{qq} + \frac{Q_{tot}}{N_e}(\mathbf{a}\cdot\boldsymbol{\epsilon}) - \bar{\eta}_q\right).
\end{aligned}
\tag{21}
$$

After the displacement, the parameters $\bar{\eta}_p$ and $\bar{D}_{pq}$ are not optimal anymore as the energy is changed due to the $\mathbf{a}$ contributions in the dipole. However, we note that the minimum energy is obtained for $\tilde{\eta}_p = \bar{\eta}_p - (Q_{tot}/N_e)(\mathbf{a}\cdot\boldsymbol{\epsilon})$ and the same $\bar{D}_{pq}$ as before. This also implies that the gradients return to zero. Using a similar line of argument we show that the orbital energies are not affected by displacement of the molecule. The dipole integrals that enter the D_{pq} gradient are affected by a displacement of the system as shown in Equation (20). However, the quantity $((\mathbf{d}\cdot\boldsymbol{\epsilon})_{pp} - \eta_p)$ is unchanged choosing $\tilde{\eta}_p$. Therefore, the Fock matrix is unaltered and the eigenvalues remain the same.

Size-extensivity in SC-QED-HF

In this section we show that the SC-QED-HF method is size-extensive.
Assume that we solved the SC-QED-HF equations for two systems A and B

$$
\begin{aligned}
\text{A system :} \quad & |\psi\rangle_A = \exp\left(-\frac{\lambda}{\sqrt{2\omega}} \sum_{p_A} \bar{\eta}_{p_A} E_{p_A p_A} (b - b^\dagger)\right) \exp(\bar{\kappa}_A) \prod_{i_A}^{n_{occ}^A} a_{i_A} |0\rangle_A \\
\text{B system :} \quad & |\psi\rangle_B = \exp\left(-\frac{\lambda}{\sqrt{2\omega}} \sum_{p_B} \bar{\eta}_{p_B} E_{p_B p_B} (b - b^\dagger)\right) \exp(\bar{\kappa}_B) \prod_{i_B}^{n_{occ}^B} a_{i_B} |0\rangle_B
\end{aligned}
\tag{22}
$$

and that their individual energies are E_A and E_B. We show here that the product $|\psi_A\psi_B\rangle$ is a stationary point for the AB system. Since $D_{p_A q_B} = 0$ for the product wave function and Equation (17) is respected for A and B

separately, the Fock and the η_p gradients are equal to

$$\frac{\partial E}{\partial D_{m_A n_A}} = \tilde{h}_{m_A n_A} Q_{m_A n_A} + \sum_{r_A s_A} \left(2\tilde{g}_{m_A n_A r_A s_A} - \tilde{g}_{m_A s_A r_A n_A}\right) D_{r_A s_A} Q_{m_A n_A r_A s_A}$$
$$+ \frac{\lambda^2}{2} \delta_{m_A n_A} \left((\mathbf{d}\cdot\boldsymbol{\epsilon})_{m_A m_A} - \eta_{m_A}\right)^2$$
$$- \lambda^2 D_{n_A m_A} \left((\mathbf{d}\cdot\boldsymbol{\epsilon})_{m_A m_A} - \eta_{m_A}\right)\left((\mathbf{d}\cdot\boldsymbol{\epsilon})_{n_A n_A} - \eta_{n_A}\right)$$

$$\frac{\partial E}{\partial D_{m_A n_B}} = \frac{\partial E}{\partial D_{n_B m_A}} = 0 \tag{23}$$

$$\frac{\partial E}{\partial D_{m_B n_B}} = \tilde{h}_{m_B n_B} Q_{m_B n_B} + \sum_{r_B s_B} \left(2\tilde{g}_{m_B n_B r_B s_B} - \tilde{g}_{m_B s_B r_B n_B}\right) D_{r_B s_B} Q_{m_B n_B r_B s_B}$$
$$+ \frac{\lambda^2}{2} \delta_{m_B n_B} \left((\mathbf{d}\cdot\boldsymbol{\epsilon})_{m_B m_B} - \eta_{m_B}\right)^2$$
$$- \lambda^2 D_{n_B m_B} \left((\mathbf{d}\cdot\boldsymbol{\epsilon})_{m_B m_B} - \eta_{m_B}\right)\left((\mathbf{d}\cdot\boldsymbol{\epsilon})_{n_B n_B} - \eta_{n_B}\right) \tag{24}$$

$$\frac{\partial E}{\partial \eta_{m_A}} = \frac{\lambda^2}{\omega} \sum_{q_A} \tilde{h}_{m_A q_A} D_{m_A q_A} \left(\eta_{q_A} - \eta_{m_A}\right) Q_{m_A q_A} - \lambda^2 D_{m_A m_A} \left((\mathbf{d}\cdot\boldsymbol{\epsilon})_{m_A m_A} - \eta_{m_A}\right) \tag{25}$$
$$+ \frac{\lambda^2}{\omega} \sum_{q_A r_A s_A} \tilde{g}_{m_A q_A r_A s_A} \left(D_{m_A q_A} D_{r_A s_A} - \frac{D_{m_A s_A} D_{r_A q_A}}{2}\right) \left(\eta_{m_A} + \eta_{r_A} - \eta_{q_A} - \eta_{s_A}\right) Q_{m_A q_A r_A s_A}$$
$$+ \lambda^2 \sum_{q_A} \frac{D^2_{q_A m_A}}{2} \left((\mathbf{d}\cdot\boldsymbol{\epsilon})_{q_A q_A} - \eta_{q_A}\right) \tag{26}$$

$$\frac{\partial E}{\partial \eta_{m_B}} = \frac{\lambda^2}{\omega} \sum_{q_B} \tilde{h}_{m_B q_B} D_{m_B q_B} \left(\eta_{q_B} - \eta_{m_B}\right) Q_{m_B q_B} - \lambda^2 D_{m_B m_B} \left((\mathbf{d}\cdot\boldsymbol{\epsilon})_{m_B m_B} - \eta_{m_B}\right) \tag{27}$$
$$+ \frac{\lambda^2}{\omega} \sum_{q_B r_B s_B} \tilde{g}_{m_B q_B r_B s_B} \left(D_{m_B q_B} D_{r_B s_B} - \frac{D_{m_B s_B} D_{r_B q_B}}{2}\right) \left(\eta_{m_B} + \eta_{r_B} - \eta_{q_B} - \eta_{s_B}\right) Q_{m_B q_B r_B s_B}$$
$$+ \lambda^2 \sum_{q_B} \frac{D^2_{q_B m_B}}{2} \left((\mathbf{d}\cdot\boldsymbol{\epsilon})_{q_B q_B} - \eta_{q_B}\right), \tag{28}$$

which are all equal to zero if Equations (22) are respected. The energy of the total system is moreover equal to the sum of the energy of the two systems as shown below:

$$\begin{aligned}
E = &\sum_{p_A q_A} \tilde{h}_{p_A q_A} D_{p_A q_A} Q_{p_A q_A} + \sum_{p_B q_B} \tilde{h}_{p_B q_B} D_{p_B q_B} Q_{p_B q_B} \\
&+ \frac{\lambda^2}{2} \sum_{p_A} D_{p_A p_A} \left(d_{p_A p_A} - \eta_{p_A}\right)\left(d_{p_A p_A} - \eta_{p_A}\right) + \frac{\lambda^2}{2} \sum_{p_B} D_{p_B p_B} \left(d_{p_B p_B} - \eta_{p_B}\right)\left(d_{p_B p_B} - \eta_{p_B}\right) \\
&+ \frac{\lambda^2}{2} \sum_{p_A q_A} \left(D_{p_A p_A} D_{q_A q_A} - \frac{D_{p_A q_A} D_{q_A p_A}}{2}\right) \left(d_{p_A p_A} - \eta_{p_A}\right)\left(d_{q_A q_A} - \eta_{q_A}\right) \\
&+ \frac{\lambda^2}{2} \sum_{p_B q_B} \left(D_{p_B p_B} D_{q_B q_B} - \frac{D_{p_B q_B} D_{q_B p_B}}{2}\right) \left(d_{p_B p_B} - \eta_{p_B}\right)\left(d_{q_B q_B} - \eta_{q_B}\right) \\
&+ \frac{1}{2} \sum_{p_A q_A r_A s_A} \tilde{g}_{p_A q_A r_A s_A} \left(D_{p_A q_A} D_{r_A s_A} - \frac{D_{p_A s_A} D_{r_A q_A}}{2}\right) Q_{p_A q_A r_A s_A} \\
&+ \frac{1}{2} \sum_{p_B q_B r_B s_B} \tilde{g}_{p_B q_B r_B s_B} \left(D_{p_B q_B} D_{r_B s_B} - \frac{D_{p_B s_B} D_{r_B q_B}}{2}\right) Q_{p_B q_B r_B s_B} \\
= &E_A + E_B,
\end{aligned} \tag{29}$$

where Q_{pq} and Q_{pqrs} are defined as in Equation (15). In passing, we point out that non size-extensive effects in optical cavities can be described using the SC-QED-HF formalism by optimizing the correlation basis or by including more photons in the reference function. These aspects will be discussed and analyzed in a forthcoming publication.

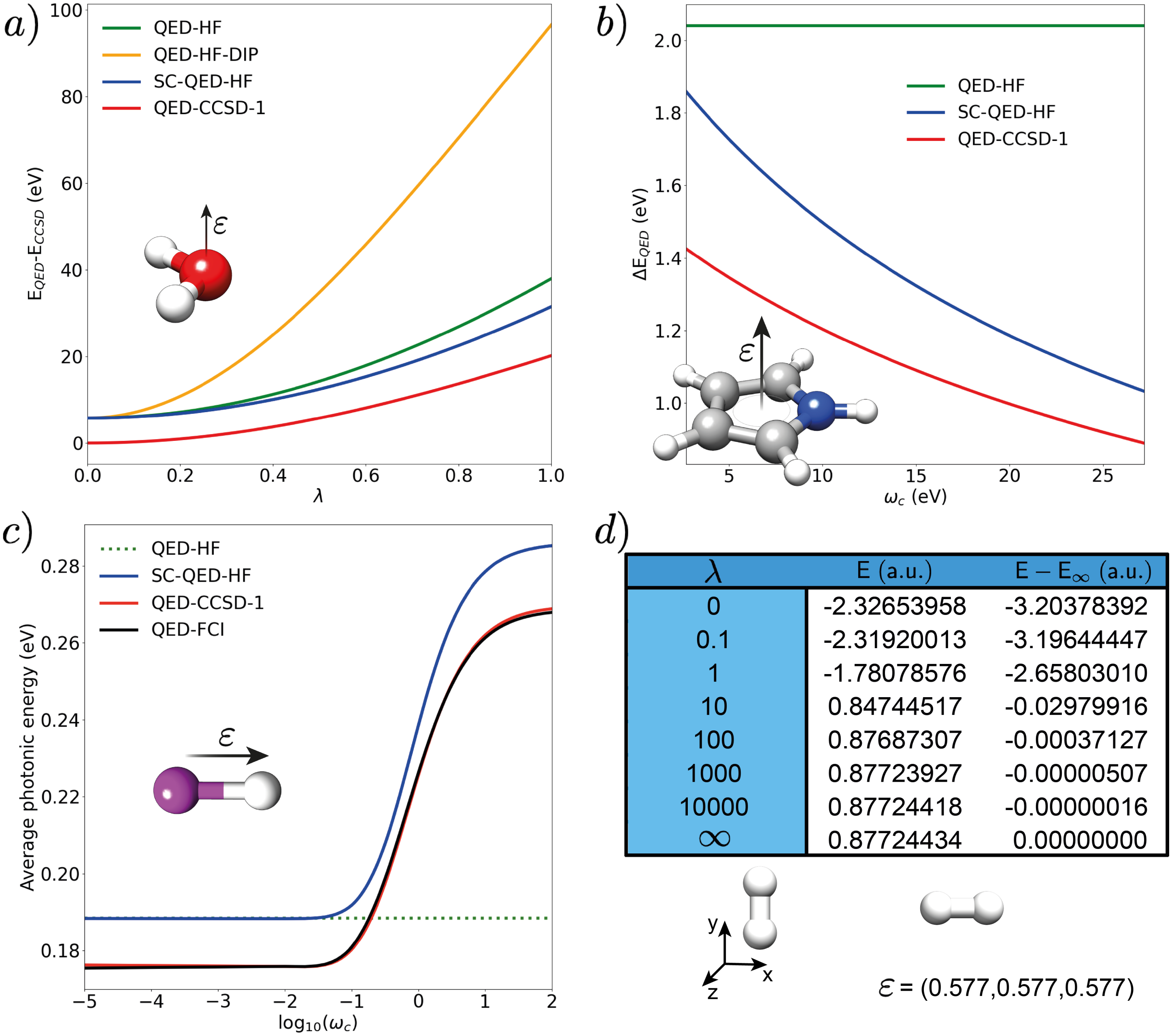

λ	E (a.u.)	$E - E_\infty$ (a.u.)
0	-2.32653958	-3.20378392
0.1	-2.31920013	-3.19644447
1	-1.78078576	-2.65803010
10	0.84744517	-0.02979916
100	0.87687307	-0.00037127
1000	0.87723927	-0.00000507
10000	0.87724418	-0.00000016
∞	0.87724434	0.00000000

Supplementary Figure 1: **Properties of the SC-QED-HF method** a) Energy dispersion of a water molecule with respect to the coupling. We notice that SC-QED-HF outperforms all the other HF based methods and shows a coupling dispersion similar to one from QED-CCSD-1. b) Energy dispersion of pyrrole with respect to the cavity frequency. The SC-QED-HF results are qualitatively correct when compared to the QED-CCSD-1 results. c) Dispersion of the photonic energy $\langle \omega b^\dagger b \rangle$. Again, the SC-QED-HF description is qualitatively correct, while a quantitative agreement with the exact solution (QED-FCI) is achieved only using a QED-CCSD-1. d) Convergence of QED-FCI and SC-QED-HF energies for increasing coupling. This table shows that as λ increases the QED-FCI energy (in atomic units) for two hydrogen molecules converges to the SC-QED-HF energy.

Supplementary Discussion

Correlation in SC-QED-HF

Comparing the QED-HF and the SC-QED-HF wave functions

$$
\begin{aligned}
|\psi\rangle_{\text{QED-HF}} &= \exp\left(-\frac{\lambda\langle \mathbf{d}\cdot\boldsymbol{\epsilon}\rangle}{\sqrt{2\omega}}(b-b^\dagger)\right)\exp(\kappa)\prod_i^{n_{occ}} a_i^\dagger \left|vac\right\rangle \otimes |0\rangle \\
|\psi\rangle_{\text{SC-QED-HF}} &= \exp\left(-\frac{\lambda}{\sqrt{2\omega}}\sum_p \eta_p E_{pp}(b-b^\dagger)\right)\exp(\kappa)\prod_i^{n_{occ}} a_i^\dagger \left|\text{vac}\right\rangle \otimes |0\rangle\,,
\end{aligned}
\tag{30}
$$

we observe that the latter includes explicit electron-photon correlation. In particular, electron-photon correlation is introduced by:

- Allowing every molecular orbital to have a different coherent state η_p;
- The orbital specific coherent state transformation is in the dipole basis rather that in the canonical basis.

To illustrate how such features influence the method performance, we compare in Supplementary Figure 1 different QED approaches. In Supplementary Figure 1a we show how the energy of a water molecule changes inside the cavity due to variations of the coupling . In particular, we compare the results obtained using QED-HF, SC-QED-HF, QED-CCSD-1 and QED-HF-DIP. In particular, QED-HF-DIP is the method obtained when the η_p parameters are not variationally optimized in Equation (9) but fixed to the eigenvalues of $(\mathbf{d}\cdot\boldsymbol{\epsilon})$:

$$
|\psi\rangle_{\text{QED-HF-DIP}} = \exp\left(-\frac{\lambda}{\sqrt{2\omega}}\sum_p (\mathbf{d}\cdot\boldsymbol{\epsilon})_{pp} E_{pp}(b-b^\dagger)\right)\exp(\kappa)\prod_i^{n_{occ}} a_i^\dagger \left|\text{vac}\right\rangle \otimes |0\rangle\,,
\tag{31}
$$

The energies obtained from QED-HF-DIP increase too fast with the coupling showing that the optimization of the η_p is needed to obtain a good approximation of the wave function. The energy dispersions obtained using QED-HF and SC-QED-HF are more similar to QED-CCSD-1, used here as a reference. Specifically, SC-QED-HF is consistently better than QED-HF.
One of the most important properties of SC-QED-HF is the energy dependence on the cavity frequency. This feature is displayed in Supplementary Figure 1b for pyrrole. We observe that the SC-QED-HF is in qualitative agreement with the one obtained with QED-CCSD-1, while for QED-HF there is no dispersion at all. Moreover, we note that the agreement becomes increasingly better as the frequency of the cavity is increased because less photons are needed to describe the wave function.
In Supplementary Figure 1c, the dispersion of the photonic energy $\langle \omega b^\dagger b\rangle$ in length gauge is computed with different methods. As expected, QED-HF does not show any dispersion at all in the frequency while the SC-QED-HF description is qualitatively correct. Quantitative agreement to the exact results (QED-FCI) is only obtained with correlated methods like QED-CCSD-1.
In Supplementary Figure 1d we show the convergence between the results for very large couplings computed with SC-QED-HF and QED-FCI in this way demonstrating that the SC-QED-HF solution is indeed exact for $\lambda \longrightarrow \infty$.

Supplementary figures

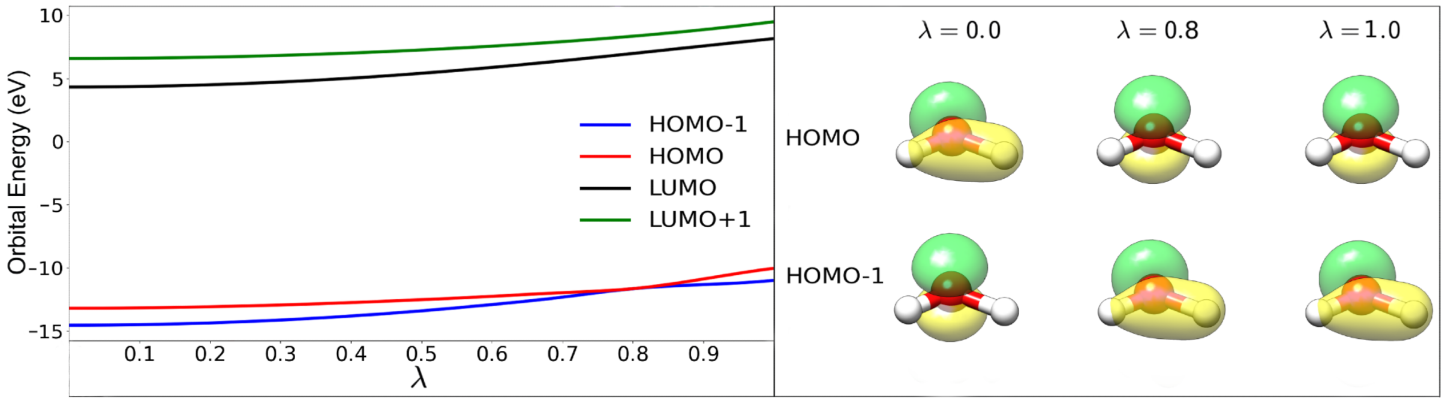

Supplementary Figure 2: **Symmetry allowed crossing between HOMO and HOMO-1 of a water molecule in an optical cavity**

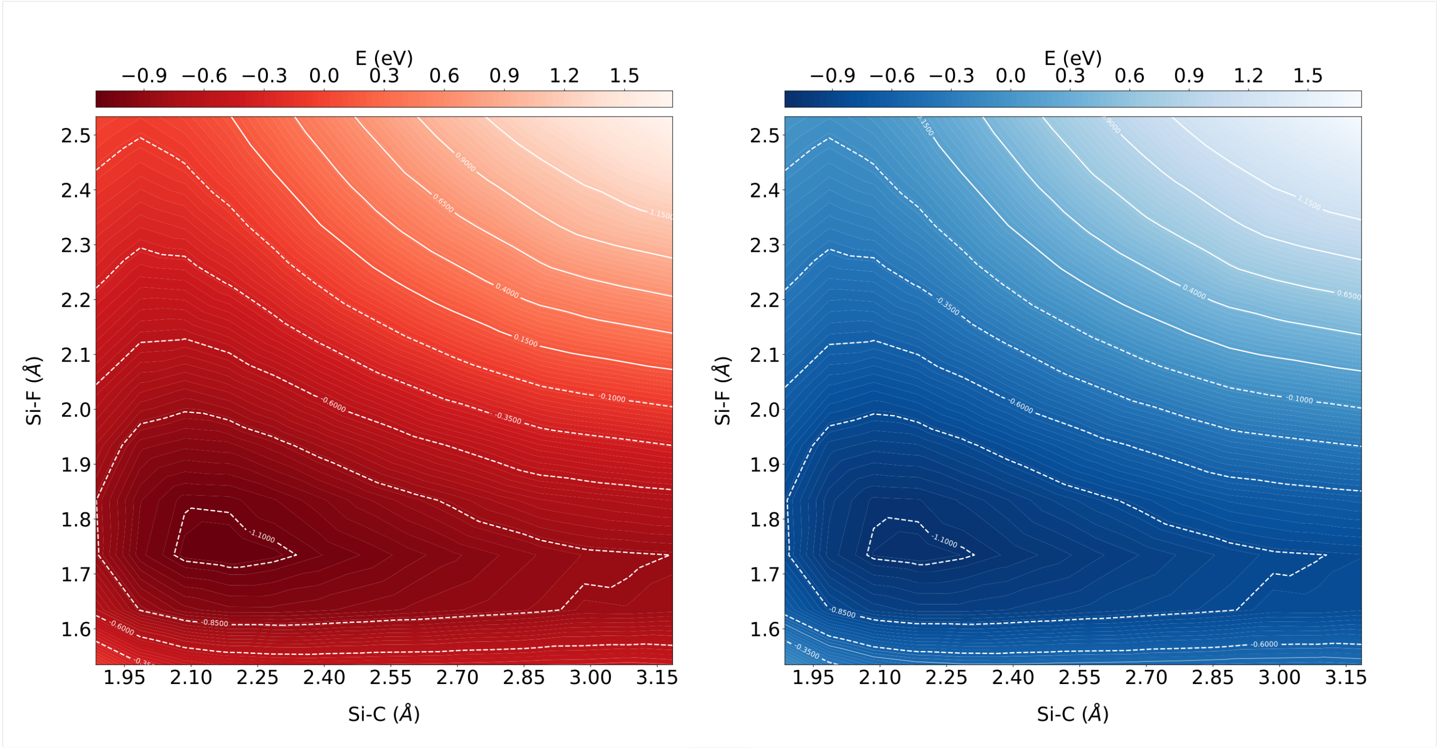

Supplementary Figure 3: **Potential energy surfaces for the TPA deprotection in TBAF inside and outside the cavity.** The two surfaces have been shifted such that the point (1.9Å, 2.5Å) has zero energy. The PES shape is not affected significantly by the presence of the cavity. The coupling is set to 0.025.

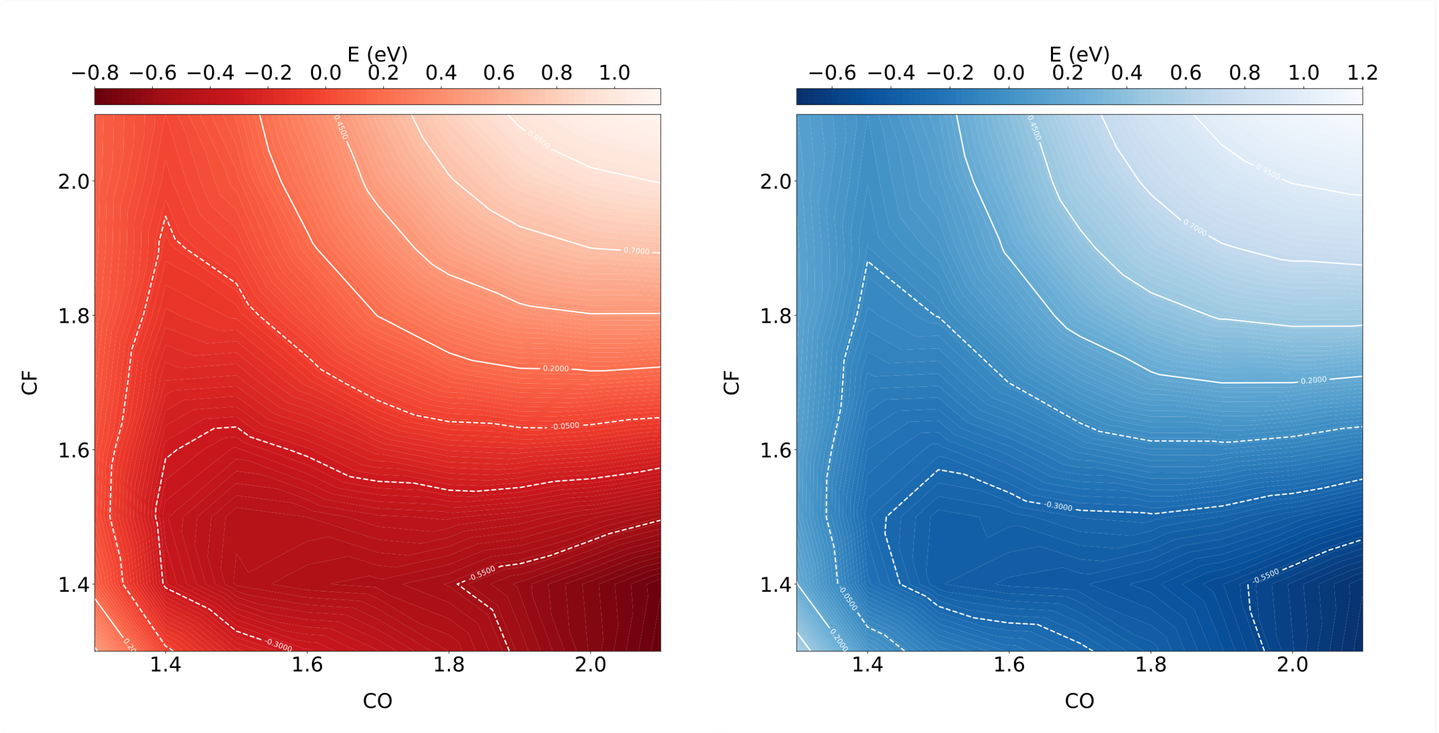

Supplementary Figure 4: **Potential energy surface for the solvolysis of PNPA inside and outside the cavity.** The two surfaces have been shifted such that the point (1.3Å, 2.1Å) has zero energy. The PES shape is not affected significantly by the presence of the cavity. The coupling is set to 0.044.
